# 

**Published:** 1904-08

**Authors:** 


					﻿CONTENTS.
ORIGINAL COMMUNICATIONS—
Study of a Case of Lateral Curvature of the Spine. A Report on an Operation for the De-
formity. By Michael Hoke, M.D., Atlanta, Ga................................239
Preventive Medicine : Its Achievements, Scope and Possibilities. By Hermann M. Briggs, M.D.,
New York............ ;.....................................................310
’The Prevention of Diarrhea, with Special Reference to Bacteriology. By Robt. W. Hynds, M.D.,
•	Atlanta, Ga...............................................................322
EDITORIAL NOTES AND COMMENTS—
The Therapeutics of Music.................................................... 329
Tax on Patent Medicines.................................................... 331
NEWS AND NOTES.:.................................................'............ 333
CONTENTS CONTINUED...............................................................   X
I
CONTENTS— CONTINUED.
SELECTIONS AND ABSTRACTS—
Medicine and Its Achievements............................... 337
The Treatment of Cancer....................................  352
“Bilious” Attacks, or Acute Intestinal Auto-toxemia......... 357
Notes on the Atlantic City Meeting.......................... 359
MEDICAL ITEMS—Continued................xviii, xx, xxii, xxiv, xxvi, xxviii, xxxv.
				

